# Chromosome-level genome assembly and annotation of the Yunling cattle with PacBio and Hi-C sequencing data

**DOI:** 10.1038/s41597-024-03066-w

**Published:** 2024-02-23

**Authors:** Zaichao Wei, Lilian Zhang, Lutao Gao, Jian Chen, Lin Peng, Linnan Yang

**Affiliations:** 1 College of Food Science and Technology, Yunnan Agricultural University, Kunming, China; 2 College of Big Data, Baoshan University, Baoshan, China; 3 College of Big Data, Yunnan Agricultural University, Kunming, China; 4 Yunnan Engineering Technology Research Center of Agricultural Big Data, Kunming, China; 5 Yunnan Engineering Research Center for Big Data Intelligent Information Processing of Green Agricultural Products, Kunming, China

**Keywords:** Data processing, Genome informatics

## Abstract

Yunling cattle is a new breed of beef cattle bred in Yunnan Province, China. It is bred by crossing the Brahman, the Murray Grey and the Yunnan Yellow cattle. Yunling cattle can adapt to the tropical and subtropical climate environment, and has good reproductive ability and growth speed under high temperature and high humidity conditions, it also has strong resistance to internal and external parasites and with good beef performance. In this study, we generated a high-quality chromosome-level genome assembly of a male Yunling cattle using a combination of short reads sequencing, PacBio HiFi sequencing and Hi-C scaffolding technologies. The genome assembly(3.09 Gb) is anchored to 31 chromosomes(29 autosomes plus one X and Y), with a contig N50 of 35.97 Mb and a scaffold N50 of 112.01 Mb. It contains 1.62 Gb of repetitive sequences and 20,660 protein-coding genes. This first construction of the Yunling cattle genome provides a valuable genetic resource that will facilitate further study of the genetic diversity of bovine species and accelerate Yunling cattle breeding efforts.

## Background & Summary

Yunling cattle, a new hybrid breed of beef cattle, was bred by the Academy of Grassland and Animal Science in Yunnan, China. As the fourth beef cattle breed with fully independent intellectual property rights bred by Chinese scientific researchers, Yunling cattle has attracted more and more attention. The cattle represents not only the first meat cattle breed bred by three-way hybridization in China, but also the first new beef cattle breed adapted to the tropical and subtropical areas of southern China^1^. Its final genetic composition is from 50% Brahman cattle, 25% Murray Grey, and 25% Yunnan Yellow cattle. With their enhanced growth and high meat production rate from Murray Grey, good reproductive capacity from Yunnan Yellow cattle, and adaptation to high temperature and high humidity conditions from Brahman, Yunling cattle have become a crucial source of beef production in China^2^. Some studies have indicated that Yunling cattle have good fattening performance, notable physical proportions, increased meat yield, favorable carcass traits, and a desirable fatty acid composition in their meat^3^. However, the molecular mechanisms that are responsible for these phenotypic variations have not yet been fully elucidated^4^. Therefore, more research is needed to understand the basis of the development of good traits in Yunling cattle.

In this paper, we constructed a chromosome-level genome of Yunling cattle by combining short reads, PacBio HiFi(high fidelity) reads, and Hi-C(High-throughput chromosome conformation capture) sequencing data. We extracted genomic DNA from heart tissue, constructed different libraries, and sequenced them using an appropriate platform. After quality filtering and trimming of the raw data, Hifiasm^5^ software was employed to assemble the genome using clean HiFi reads. To further improve the accuracy of the assembly, the assembly was refined with Nextpolish^6^ software using short reads with default parameters. Subsequently, we applied the PacBio HiFi reads and Hi-C data to generate a high-quality chromosome-level genome assembly of Yunling cattle. The final genome assembly(3.09 Gb) was anchored to 31 chromosomes, containing 1119 contigs(N50 = 35.97 Mb) and 826 scaffolds (N50 = 112.01 Mb). A total of 1.62 Gb of repeat sequences were identified, representing 52.26% of the total genome, of which 99.80% were classified as known repeat elements. In addition, structural annotation of the genome yielded 20,660 genes, of which 92.8% (19,172) could be functionally annotated with at least one of the five protein databases (NR, SwissProt, KOG, GO and KEGG). The Yunling cattle genome assembled in this study provides a valuable genetic resource for future efforts to study Yunling cattle and further comparative analysis of genome biology among bovine species to promote breeding research.

## Methods

### Sample collection

A four-year-old male Yunling cattle from the Chuxiong JingDa Farm in Chuxiong City, Yunnan Province, was used for genome sequencing and assembly. Pectoralis profundus muscle, Cervical part of the trapezius muscle, Latissimus dorsi muscle, Internal abdominal oblique muscle, Gluteobiceps muscle, lung, spleen, liver, and heart tissues were collected and rapidly frozen in liquid nitrogen. Heart tissues were used for DNA sequencing for genome assembly, while all tissues were used for transcriptome sequencing.

### library construction and sequencing

Genomic DNA from heart tissue was extracted using the standard phenol-chloroform extraction method for DNA sequencing library construction. The integrity of the genomic DNA molecules was checked using agarose gel electrophoresis.

In addition to that two types of libraries were constructed,the BGISEQ DNBSEQ-T7 platform and the PacBio Sequel II platform (CCS mode) were applied for genomic sequencing to generate short and HiFi genomic reads, respectively. For the BGISEQ DNBSEQ-T7 platform (Shenzhen, Guangdong, China), a short-read paired-end sequencing library with an insert size of 350 bp was prepared according to the protocol provided by the manufacturer and sequenced using the BGISEQ DNBSEQ-T7 platform at GrandOmics Biosciences Co., Ltd. (Wuhan, China). This resulted in accurate short reads of 161.89 Gb (approximately 64x coverage of the estimated genome size, Table 1). These reads were further cleaned using the fastp^7^ utility. Adapter sequences and reads containing more than 10% N bases or low quality bases (≤5) were removed from the raw sequencing data. After filter, 150.59 Gb of cleaned data were retained for the subsequent analysis. To attain adequate sequencing data for genome assembly, we constructed two 15 kb DNA libraries utilizing the extracted DNA and the standard Pacific Biosciences (PacBio, Menlo Park, CA) protocol, and fragments were chosen via the Blue Pippin Size-Selection System (Sage Science, MA, USA). The two libraries were sequenced using Single-Molecule Real-Time (SMRT) cells with the PacBio Sequel II platform (CCS mode) in GrandOmics Biosciences Co., Ltd.(Wuhan, China). After removing adaptors, we obtained 61.81 Gb of HiFi subreads (Table 1) for genome assembly. The genome sequencing data used for the subsequent genome assembly are summarized in Table 1.

**Table 1:** Sequencing data used for the Yunling cattle genome assembly.

Library resource	Sequencing platform	Insert size	Raw data(Gb)	Sequence coverage (X)
genome	BGISEQ DNBSEQ-T7	350 bp	161.89	64
genome	PacBio SEQUELII	15Kb	61.8	25
Hi-C	BGISEQ DNBSEQ-T7	350 bp	427.1	170
transcriptome	BGISEQ DNBSEQ-T7	350 bp	121.67	−

Note that the sequence coverage values were calculated based on the genome size estimated by the Kmer-based method.

For Hi-C sequencing, we constructed a library based on the standard protocol of Belton *et al*. with some modifications^8^. Briefly, heart tissues were ground into small pieces and then vacuum infiltrated in a nuclei isolation buffer that was supplemented with 2% formaldehyde. Crosslinking was halted by the addition of glycine and further vacuum infiltration. Fixed tissues were ground into powder before being re-suspended in a nuclei isolation buffer to obtain a nuclei suspension. The purified nuclei was digested with 100 units of DpnII and labeled by incubation with biotin-14-dCTP.Biotin-14-dCTP from non-ligated DNA ends was eliminated due to the exonuclease activity of T4 DNA polymerase. The ligated DNA was fragmented into 300–600 bp fragments, followed by blunt-end repair and A-tailing. The DNA was then purified through biotin-streptavidin-mediated pull-down. Finally, the Hi-C libraries were quantified and sequenced via the BGISEQ DNBSEQ-T7 platform at GrandOmics Biosciences Co., Ltd.(Wuhan, China).

RNA sequencing was conducted for the generation of transcriptome data to predict gene models. To incorporate as many tissue-specific transcripts as possible, various tissues were collected, as indicated in the sample collection section. TRIzol reagent (Invitrogen, USA) was used to extract separately RNA from all collected tissues, including Pectoralis profundus muscle, Cervical part of the trapezius muscle, Latissimus dorsi muscle, Internal abdominal oblique muscle, Gluteobiceps muscle, lung, spleen, liver, and heart tissues of Yunling cattle, according to the manufacturer’s protocol. RNA quality was checked using a NanoDrop ND-1000 spectrophotometer (Labtech, Ringmer, UK) and a 2100 Bioanalyzer (Agilent Technologies, CA, USA). Next, RNA-Seq libraries were prepared using the MGIEasy RNA Sample Prep Kit (BGI, China) and sequenced using the BGISEQ DNBSEQ-T7 platform at GrandOmics Biosciences Co., Ltd. (Wuhan, China). In total, 121.67 Gb of short-read RNA-seq data were obtained (Table 1). These RNA-seq data were used for whole-genome protein-coding gene prediction.

### De novo assembly of the Yunling cattle genome

To understand the genomic characteristics of Yunling cattle, k-mer analysis using short paired-end reads was performed prior to genome assembly to estimate the genome size and heterozygosity. In brief, the quality filtered reads were subjected to a 27-mer frequency distribution analysis using the KMC^9^ and GenomeScope^10^ software. The following equation was used to estimate the genome size of the Yunling cattle: G = K-num/K-depth (where K-num is the total number of 27-mers, K-depth denotes the k-mer depth, and G represents the genome size). The genome size of the Yunling cattle was estimated from the frequency distribution to be 2.8 Gb.

For de novo genome assembly, after obtaining the HiFi long reads, the genome was de novo assembled into a preliminary assembly using Hifiasm with HiFi long reads. To further improve the accuracy of the assembly, the preliminary assembly was refined with Nextpolish using short reads with default parameters through 4 rounds. Finally, the genome size was 3.10 Gb, composed of 1,129 contigs, and the contig N50 was 38.85 Mb (Table 2).The detailed statistical results are shown in the Table 2.

**Table 2:** Assembly statistics for the Yunling cattle.

Stat Type	Polished Genome		Hi-C-data-based Chromosome-level Genome			
	Contig Length(bp)	Contig Number	Contig Length(bp)	Contig Number	Scaffold Length(bp)	Scaffold Number
N50	38854192	25	35968234	26	112006702	12
N60	27472984	35	27094936	36	86014837	16
N70	16203554	50	16203554	51	79453489	20
N80	6971123	79	6971123	81	64467655	24
N90	1970719	159	2004949	159	45043064	30
Longest	105822721	1	105822721	1	158025302	1
Total	3,100,702,976	1129	3092942564	1119	3092971864	826
Length > = 1 kb	3,100,702,976	1129	3092942564	1119	3092971864	826
Length > = 2 kb	3,100,702,976	1129	3092942564	1119	3092971864	826
Length > = 5 kb	3,100,702,976	1129	3092942564	1119	3092971864	826

### Hi-C assisted scaffolding

**Fig. 1:**
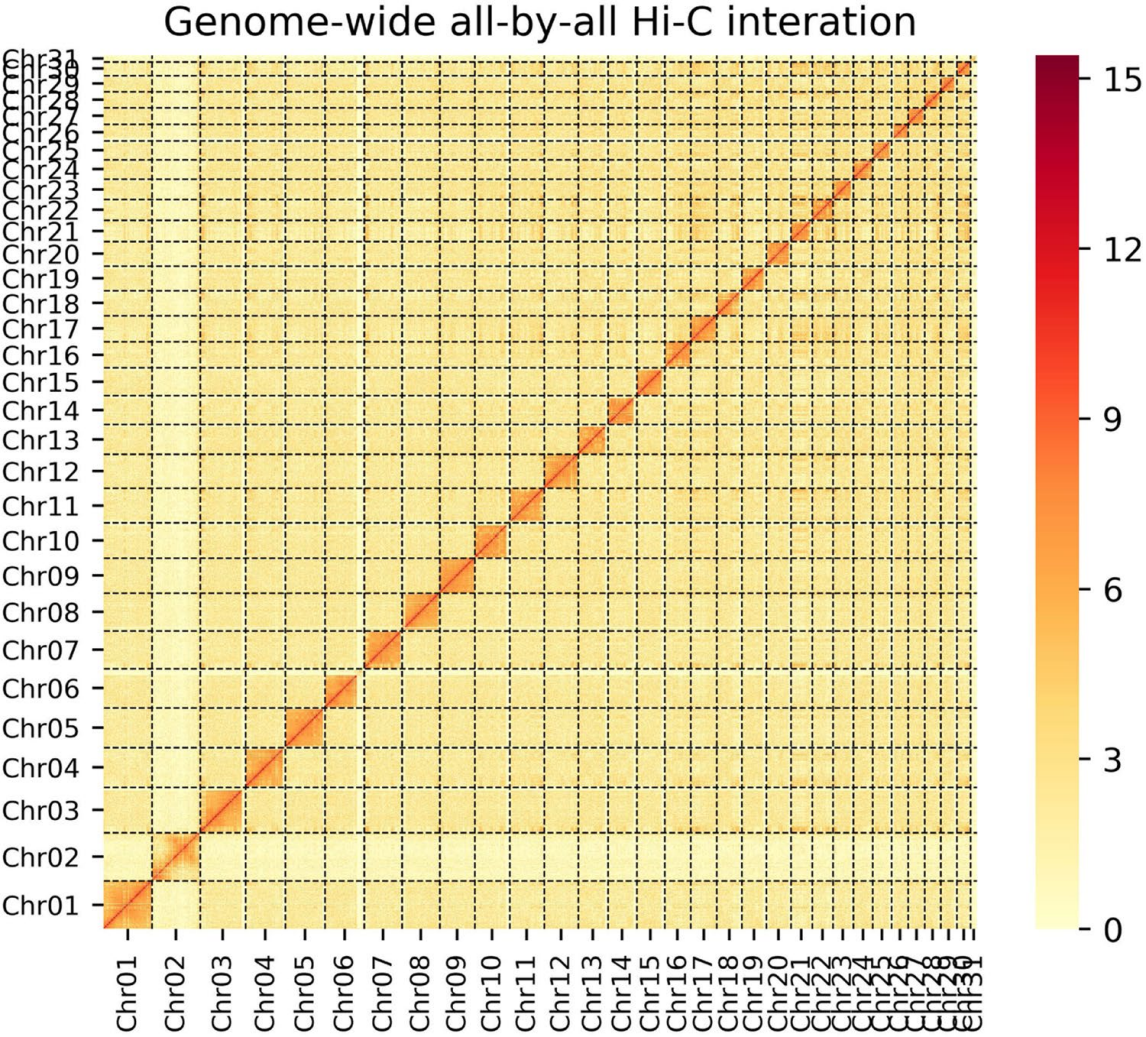
Hi-C interaction heatmap for Yunling cattle genome.

The quality control of the Hi-C raw data was carried out with the HiC-Pro^11^ software. First, low quality sequences (quality scores <20), adaptor sequences and sequences shorter than 30 bp were filtered out using fastp. Second, the clean paired-end reads were mapped to the assembly using bowtie2^12^ (-end-to-end–very-sensitive -L 30) to obtain the unique mapped paired-end reads.Third,valid interaction paired reads were identified from unambiguously mapped paired-end reads and retained by HiC-Pro for further analysis. HiC-Pro filters out invalid read pairs such as dangling-end, self-cycle, re-ligation and dumped products. Then the scaffolds were further clustered, ordered, and oriented onto chromosomes by LACHESIS^13^. Finally, Juicebox^14^ was used to manually correct large-scale inversions and translocations to obtain the final pseudochromosomes. As a result, the chromosome-level genome assembly was 3.09 Gb in length with contig and scaffold N50 values of 35.97 Mb and 112.01 Mb, respectively (Table 2). A heatmap was drawn to illustrate the interaction of each chromosome(Fig. 1).

To evaluate the quality of the assembled genome, the completeness and accuracy were assessed via BUSCO (Benchmarking Universal Single Copy Orthologs)analysis and short-read mapping. The completeness of the assembled Yunling cattle genome was assessed by using BUSCO^15^ with the mammalia_odb10 database. We found that 8,837(95.78%) of the 9,226 conserved single-copy genes in mammals were present in our assembly (Table 3). We also aligned NGS short reads to the genome and found that 99.03% of the reads were reliably aligned, showing a high mapping ratio for the short-read sequencing data.

**Table 3:** BUSCO assessment results.

Type	Number	Percent(%)
Complete BUSCOs (C)	8,837	95.78
Complete and single-copy BUSCOs (S)	8,580	93
Complete and duplicated BUSCOs (D)	257	2.79
Fragmented BUSCOs (F)	114	1.24
Missing BUSCOs (M)	275	2.98
Total BUSCO groups searched	9,226	100

### Repetitive element identification

We first annotated the tandem repeats by employing the software GMATA^16^ and Tandem Repeats Finder(TRF)^17^. GMATA identified the simple repeat sequences (SSRs), while TRF detected all tandem repeat elements across the entire genome. Transposable elements (TEs) in the genome of Yunling cattle were then identified using both ab initio and homology-based methods. Briefly, an ab inito repeat library for genome of Yunling cattle was initially predicted using MITE-Hunter^18^ and RepeatModeler^19^ with default settings.The obtained library was aligned with TEclass Repbase (http://www.girinst.org/repbase)^20^ for the purpose of classifying the type of every repeat family. To identify repeats across the genome, RepeatMasker^21^ tool was used to search for both known and novel TEs by mapping sequences against the de novo repeat library and Repbase TE library.Overlapping transposable elements of identical repeat classes were collated and merged. A total of 1.62 Gb repeat sequences which represent 52.26% of the entire genome, have been identified. Among these sequences, 99.80% have been classified as known repeat elements, as shown in Table 4.

**Table 4:** Summary statistics of repetitive elements in the assembled Yunling cattle genome.

Class	Order	Number of elements	Length of sequence (bp)	Percentage of sequence (%)
Class I	LINE	1,945,095	770,980,935	24.93
	SINE	1,365,442	220,291,436	7.12
	LTR	746,767	197,751,834	6.39
		4,057,304	1,189,024,205	38.44
Class II	DNA	641,080	75,165,372	2.43
	MITE	49,103	14,714,303	0.48
	RC	17,908	913,871	0.03
		708,091	90,793,546	2.94
Total TEs		4,765,395	1,279,817,751	41.38
Tandem Repeats	tandem_repeat	126,857	8,704,879	0.28
	SSR	194,553	2,232,218	0.07
		321,410	10,937,097	0.35
Other		169,715	317,753,347	10.27
Simple repeats		20,720	1,580,261	0.05
Unknown		17,370	6,391,973	0.21
Low complexity		121	27,734	0
Total Repeats		5,294,731	1,616,508,163	52.26

### Protein-coding genes prediction

Three independent approaches, including ab initio prediction, homology search, and reference guided transcriptome assembly, were used for gene prediction in a repeat-masked genome, resulting in 20,660 genes (Table 5). In detail, the GeMoMa^22^ software was utilised to align homologous peptides from related species to the assembly and infer the gene structure information. For RNA seq-based gene prediction, filtered mRNA-seq reads were aligned to the reference genome using STAR^23^ with default parameters. The transcripts were assembled by using stringtie^24^ and PASA^25^ was used to predict open reading frames (ORFs). For the de novo prediction, the RNA-seq reads were assembled de novo using StringTie and analyzed with PASA, resulting in the generation of a training set. Augustus^26^ with default parameters was then used for ab initio gene prediction on the training set. Finally, EVidenceModeler (EVM)^25^ was utilized to generate an integrated gene set, of which genes with TE were eliminated using TransposonPSI^27^ package (http://transposonpsi.sourceforge.net/) and the miscoded genes were further removed. Untranslated regions (UTRs) and alternative splicing regions were identified via PASA based on RNA-seq assemblies. We kept the longest transcripts for every locus, and regions outside of the ORFs were labelled as UTRs. The mean transcript length and coding sequence size were 41,167.48 and 1,604.59 bp, respectively, with an average of 9.32 exons per gene. Additionally, the average exon and intron lengths were 172.2 and 4,756.27 bp, respectively (Table 5).

**Table 5:** Summary statistics of predicted protein-coding genes of Yunling cattle.

Species	Total number of genes	Average transcript length(bp)	Average CDS length(bp)	Average exons number per gene	Average exon length(bp)	Average intron length(bp)
*Yunling cattle*	20,660	41,167.48	1,604.59	9.32	172.2	4,756.27

### Gene function annotation

**Fig. 2:**
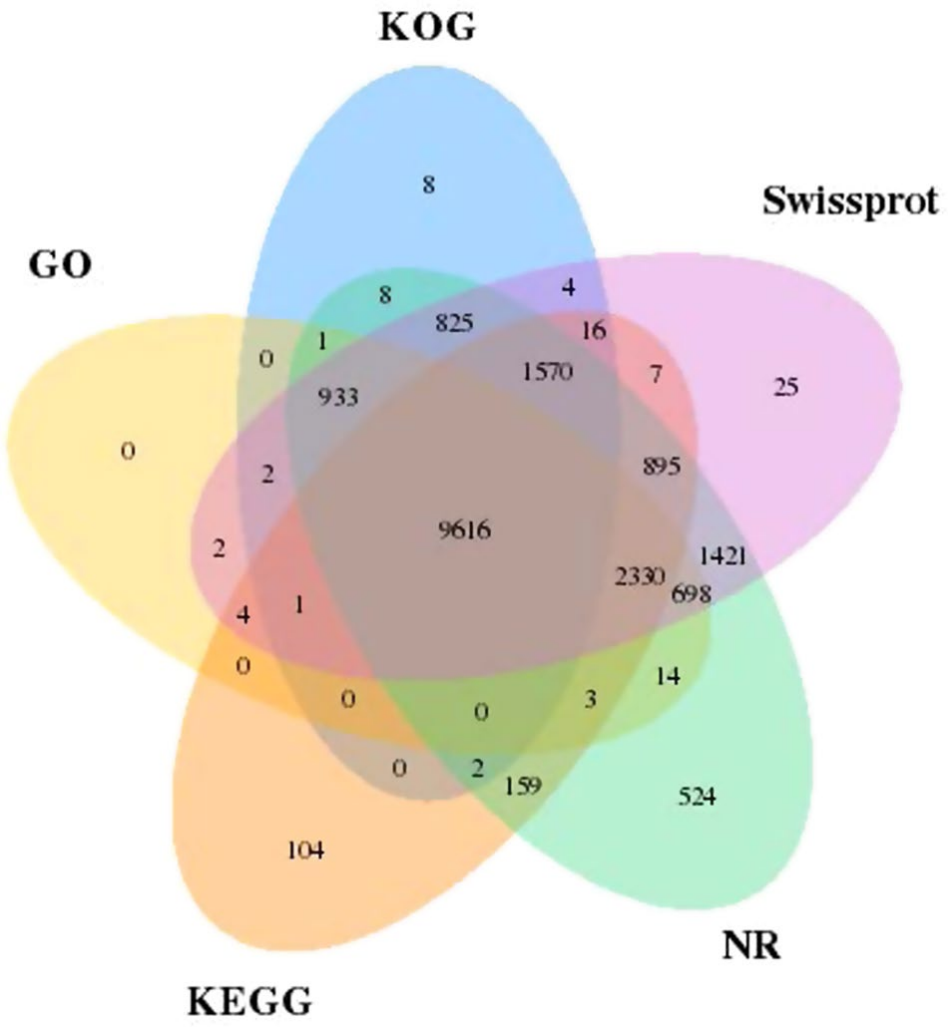
Venn diagram of annotation results for each database.

Gene functions, motifs and protein domains were determined through comparison with public databases, including SwissProt, NR (Non-Redundant Protein Database), KEGG (Kyoto Encyclopedia of Genes and Genomes), KOG (Eukaryotic Orthologous Groups of proteins) and GO (Gene Ontology). The InterProScan^28^ program with default parameters was used to identify putative domains and GO terms of genes. For the other four databases, Blastp was used to compare the EvidenceModeler-integrated protein sequences against the four well-known public protein databases with an E-value cutoff of 1e−05 and the results with the lowest E-value hit were retained. Results from the five database searches were concatenated. A total of 19,172 genes (92.80% of the predicted protein-coding genes) were annotated using the above multiple databases. Specifically, approximately 88.81%,91.96%, 71.19%, 62.86%, and 65.85% were annotated in SwissProt, NR, KEGG, KOG, and GO, respectively (Table 6, Fig. 2).

**Table 6:** Number of predicted genes of Yunling cattle functionally annotated by using indicated databases.

Type		Number	Percent (%)
Annotation	Swissprot	18,349	88.81
	KEGG	14,707	71.19
	KOG	12,986	62.86
	GO	13,604	65.85
	NR	18,999	91.96
Total	Annotated	19,172	92.8
	Total Gene	20,660	—

### Annotation of non-coding RNAs (ncRNAs)

To obtain the ncRNA (non-coding RNA), two strategies were used: searching against database and prediction with model. Transfer RNAs (tRNAs) were identified through the use of tRNAscan-SE^29^ with parameters specific to eukaryotes. MicroRNA, rRNA, small nuclear RNA, and small nucleolar RNA were identified by using Infernal cmscan to search the Rfam^30^ database. The rRNAs and their subunits were predicted using RNAmmer^31^. The predicted non-coding genes include 891 miRNAs, 259,398 tRNAs, 3,659 rRNAs, and 737 snRNAs in the Yunling cattle genome (Table 7).

**Table 7:** Summary statistics of Non-coding RNA annotation results.

Type		Copy Number	Average Length(bp)	Total Length(bp)	Percentage of sequence(%)
rRNA (3,659)	18 S	53	1,889.38	100,137	0.0032
	28 S	51	7,710.67	393,244	0.0127
	5.8 S	55	152.27	8,375	0.0003
	5 S	3,500	116.66	408,293	0.0132
miRNA (3,219)	snRNA	737	113.1	83,358	0.0027
	miRNA	891	79.85	71,143	0.0023
	spliceosomal	1,164	114.52	133,300	0.0043
	other	427	180.83	77,213	0.0025
Regulatory	cis-regulatory elements	318	67.69	21,525	0.0007
tRNA	tRNA	259,398	73.27	19,004,893	0.6145

## Data Records

The DNA and RNA sequencing data were submitted to the NCBI Sequence Read Archive (SRA) database under the SRA IDs: SRR24831383^32^, SRR24831384^33^, SRR24831385^34^, SRR24831386^35^, SRR24831387^36^, SRR24831388^37^, SRR24831389^38^, SRR24831390^39^, SRR24831391^40^, SRR24831392^41^, SRR24831393^42^, SRR24831394^43^ and SRR24831395^44^, which is associated with the BioProject accession number PRJNA978937. The assembled draft genome of Yunling cattle have been deposited at the NCBI GenBank (https://identifiers.org/ncbi/insdc.gca:GCA_034097375.1^45^). The annotation results of repeated sequences, gene structure and functional prediction have been deposited at the Figshare database (10.6084/m9.figshare.23391614^46^).

## Technical Validation

### Quality assessment of the genome assembly

In the present research work, a high-quality chromosome-scale genome assembly of the Yunling cattle was constructed by combining PacBio Hifi sequencing, short reads sequencing, and chromosome conformation capture (Hi-C) anchoring, which resulted in a genome approximately 3.09 Gb in length with contig and scaffold N50 values of 35.97 Mb and 112.01 Mb, respectively (Table 2). Contigs were scaffolded into 31 superscaffolds, accounting for 99.90% of the total genome size. As shown in the Hi-C heatmap (Fig. 1), the 31 superscaffolds in the Yunling cattle genome could be distinguished and perfectly represented by 31 chromosomes.

To evaluate the completeness of our assembly, we carried out BUSCO(Benchmarking Universal Single Copy Orthologs) and CEGMA^47^ (Core Eukaryotic Gene Mapping Approach) analyses. BUSCO results indicated that 8,837(95.78%) of the 9,226 conserved single-copy genes in mammals were present in our assembly, of which 8,580 were single, 257 were duplicated, and 114 fragmented matches (Table 3). CEGMA results indicated 237(95.56%) core genes of the 248 core eukaryotic genes were present in our assembly, of which 231(93.15%) were complete, It shows that the core gene in the genome is relatively complete.

**Fig. 3:**
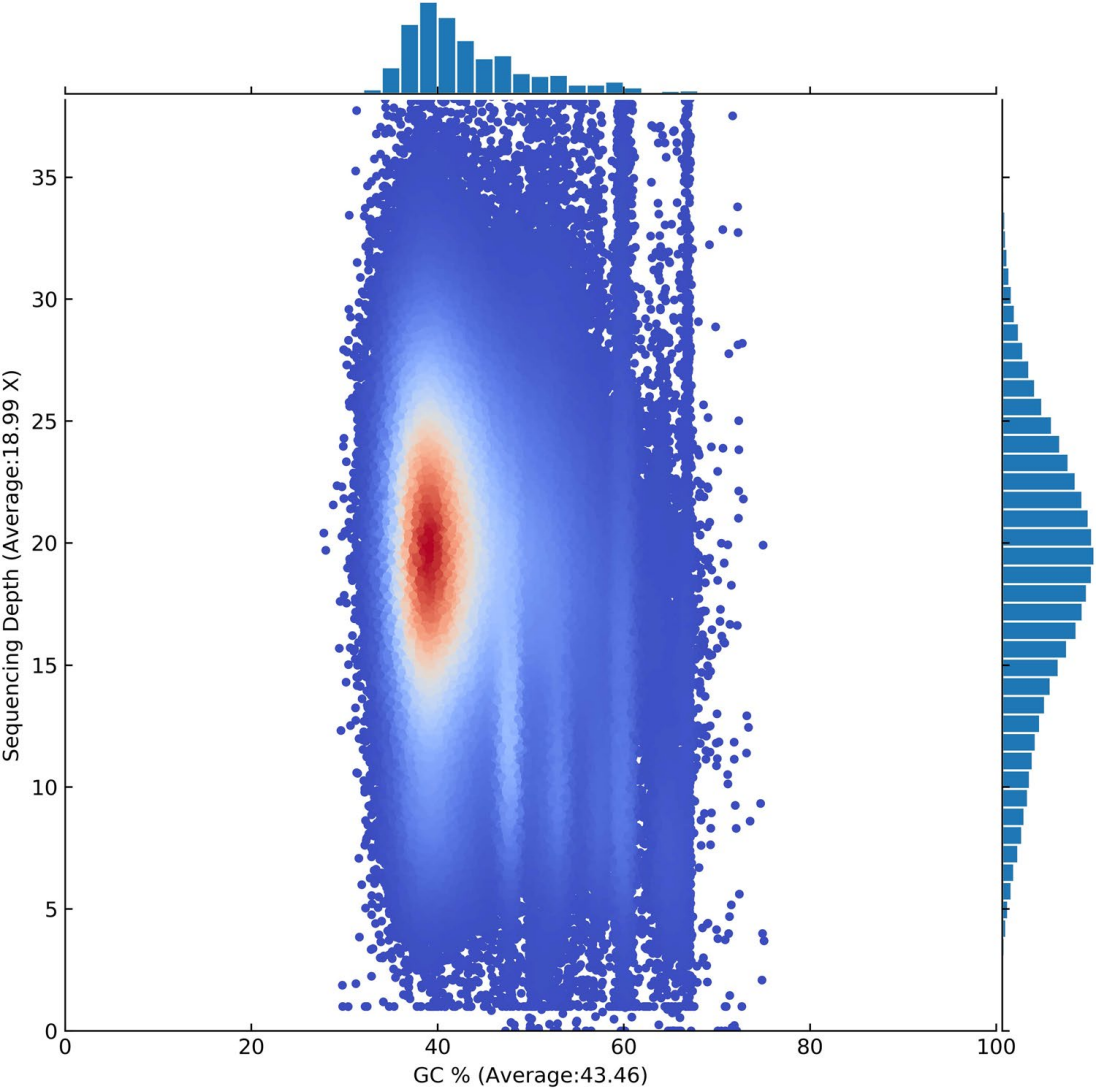
The distribution of GC depth of the genome. Note that the horizontal axis represents GC content, and the vertical axis represents depth. These two values are sequentially calculated using a 10Kb window.

To evaluate the accuracy of the assembly, all the short paired-end reads were mapped to the assembled genome using BWA (Burrows-Wheeler Aligner)^48^ and the mapping rate as well as genome coverage of sequencing reads were assessed using SAMtools^49^, we found more than 93.72% of the genome had >20-fold coverage, indicating high accuracy at the nucleotide level. Besides, the base accuracy of the assembly was calculated with bcftools^50^, the base accuracy of genomic is 99.999533% (Depth > = 5X).The results of GC-Depth analysis of the genome were shown in the figure (Fig. 3). The results show that the GC content is distributed in 20–40%, and the sequencing depth is concentrated in the 20–25X region, indicating that there is no exogenous pollution in the genome. These results have suggested our assembly has high quality and is quite complete.

### Gene prediction and annotation validation

Three independent approaches, including ab initio prediction, homology search, and reference guided transcriptome assembly, were used for gene prediction in a repeat-masked genome. The EVM^25^ software was used to integrate the gene prediction results and generate a consensus gene set. In addition, the functional annotation of these predicted genes revealed that 92. 8% (genes = 19,172) of them could be assigned to at least one functional term (Table 6, Fig. 2). These findings strongly suggest that the annotated gene set of the Yunling cattle genome is quite complete.

## Data Availability

The software versions, settings and parameters used are described below. No custom code was used during this study for the curation and/or validation of the dataset. Hifiasm v0.16.0: -t 32 Nextpolish v1.2.4: rerun = 4, gs_options = -max_depth 100 -bwa fastp v0.21.0: -n 0 -f 5 -F 5 -t 5 -T 5 -q 20 KMC v3.2.1: -k27 -t64 -m512 -ci1 -cs1000000 GenomeScope v1: 27 150 output HiC-Pro v2.8.1: -c config-hicpro.txt -i -o bowtie2 v2.3.2: -end-to-end --very-sensitive -L 30 LACHESIS v1: CLUSTER_MIN_RE_SITES = 100, CLUSTER_MAX_LINK_DENSITY = 2.5, CLUSTER NONINFORMATIVE RATIO = 1.4, ORDER MIN N RES IN TRUNK = 60, ORDER MIN N RES IN SHREDS = 60 Juicebox v 1.11.08: default BUSCO v4.0.5: -l mammalia_odb10 -o result -m genome -c 10 -f GMATA v2.2: default TRF v 4.07b: 2 7 7 80 10 50 500 -f -d -m MITE-Hunter: -n 20 -P 0.2 -c 3 RepeatModeler v1.0.11: -engine wublast RepeatMasker v1.331: nolow -no_is -gff -norna -engine abblast -lib lib GeMoMa v1.6.1: default STAR v2.7.3a: default Stringtie v1.3.4d: default PASA v2.3.3: -c Assembly.config -C -R -g genome.fasta -T -u trans.fasta -t trans.clean.fasta -f fl.acc --CPU 32 --ALIGNERS gmap Augustus v3.3.1: default EVidenceModeler v1.1.1: --segmentSize 1000000 --overlapSize 100000 InterProScan v 5.32: default Blastp v2.7.1: -e 1e-5 tRNAscan-SE v2.0: --thread 16 -E -I Infernal v1.1.2: default RNAmmer v1.2: -S euk -m lsu,ssu,tsu -gff CEGMA v 2–2.5: --genome genome.fasta --vrt --mam Bwa v0.7.15: mem -t 32 samtools v2.17: depth Bcftools v1.8.0: default
